# Vaccine therapy for pediatric high-grade glioma: current landscape, challenges, and future directions

**DOI:** 10.1007/s11060-025-05403-4

**Published:** 2026-01-08

**Authors:** Stuart D. Harper, Jacob A. Alderete, Shivani Baisiwala, Bianca H. Bergsneider, Linda M. Liau, Anthony C. Wang

**Affiliations:** 1 Department of Neurosurgery, University of California Los Angeles, Los Angeles, California USA; 2 Warren Alpert Medical School, Brown University, Providence, Rhode Island, USA

**Keywords:** Pediatric high-grade glioma, Vaccine immunotherapy, Glioma-associated antigens, Peptide vaccines, Neoantigen vaccines, mRNA vaccines

## Abstract

**Background:**

Pediatric high-grade gliomas (pHGG) are among the most aggressive childhood brain tumors, with limited treatment options and poor prognosis. Vaccine-based immunotherapy offers a promising strategy by leveraging tumor-specific or associated antigens to stimulate durable anti-tumor immune responses with minimal toxicity.

**Discussion:**

This review outlines the scientific rationale for vaccine therapies in pHGG, detailing key targets such as glioma-associated antigens (EphA2, IL-13Rα2, survivin), driver mutation–derived neoantigens (H3.3K27M, TP53, IDH1), and viral antigens (CMV pp65). We evaluate current vaccine platforms, including peptide vaccines, dendritic cell vaccines, mRNA-based vaccines, and neoantigen-personalized approaches, highlighting early-phase clinical trial results that demonstrate safety and immunogenicity. Despite encouraging preliminary data, several challenges hinder clinical translation, including the distinct immune environment in the central nervous system, intratumoral heterogeneity, low mutational burden, immunosuppressive microenvironments, steroid use, and logistical hurdles in vaccine production and trial design. Future research must address these barriers through optimized antigen selection, combinatorial therapies, novel delivery systems, and pediatric-specific immune profiling.

**Conclusion:**

With continued multidisciplinary collaboration, vaccine therapies may emerge as a meaningful addition to the therapeutic arsenal for children with pHGG.

## Introduction

Pediatric high-grade gliomas (pHGG) represent the most aggressive and lethal of the childhood brain tumors. Prognosis remains dismal, with median survival less than 24 months in 70–90% of patients, and less than 1 year in most subtypes [1–5]. The current standard of care, which includes maximal safe surgical resection and radiotherapy, has limited efficacy due to the infiltrative nature of these tumors, their resistance to cytotoxic agents, and the frequent involvement of eloquent or surgically inaccessible brain regions such as the brainstem [1, 4–6].

Over the past decade, molecular profiling has revolutionized the classification of pHGG, distinguishing them from adult gliomas and uncovering key oncogenic drivers unique to the pediatric population. This shift from reliance upon pathologic appearance to molecular identity is reflected in the most recent 2021 WHO guidelines, which heavily depend upon direct tissue sequencing for tumor classification [7, 8]. A growing understanding of the key driver mutations in pHGG, such as H3 K27M in diffuse midline glioma, H3 K27-altered (DMG), and H3 G34R/V in diffuse hemispheric glioma, H3 G34-mutant (DHG), has not only improved diagnostic precision but also opened new avenues for targeted therapies [1, 7]. Nevertheless, translating these insights into effective treatments has remained challenging, particularly given the unique biology and immunology of pediatric brain tumors.

Immunotherapy has emerged as a promising strategy in oncology, with dramatic successes in hematologic malignancies [9, 10] and select adult solid tumors [11, 12]. However, its application in pediatric neuro-oncology has lagged, constrained by factors such as the distinct nature of the immune system within the central nervous system (CNS), the immune-suppressive tumor microenvironment (TME), and the low mutational burden characteristic of pHGG [6, 13–16]. Recent landmark trials utilizing chimeric antigen receptor (CAR) T-cell therapy have demonstrated meaningful biological activity and early clinical responses, sparking increasing interest in the potential of immunotherapy for pHGG. BrainChild-03 improved median survival for 21 DIPG patients to 19.8 months utilizing repeated intracerebroventricular (ICV) B7-H3 CAR T-cell dosing with only one dose-limiting toxicity [17]. Another phase-I clinical trial (NCT04196413) employed single-dose IV and subsequent ICV infusions of GD2-CAR T-cells in lymphodepleted DIPG or spinal DMG patients, and identified volumetric reductions in 7 of 11 patients receiving therapy [18]. Notably, three patients on the higher dosing regimen developed dose-limiting cytokine release syndrome, with additional instances of tumor inflammation-associated neurotoxicity (TIAN) or immune effector cell acute neurotoxicity syndrome (ICANS). These milestone studies highlight the promising therapeutic potential, as well as the logistical and safety hurdles still to be addressed, of CAR T-cell therapy for pHGG.

While adoptive cellular therapies including CAR T-cells have provided important data to validate that the immune system can indeed be therapeutically mobilized against pHGG, these ongoing challenges may underscore the need for complementary immunotherapeutic modalities. Anti-tumor vaccine strategies represent a particularly attractive approach due to their relatively non-invasive administration and more favorable toxicity profile compared to other immunotherapeutic modalities [19, 20]. Vaccines aim to stimulate the patient’s immune system to recognize tumor-associated antigens (TAA), thereby enabling a durable anti-tumor response with minimal off-target effects and without the need for direct cellular replacement. In early-phase trials, some vaccine platforms have shown encouraging safety and immunogenicity in children with high-grade gliomas [21–26]. Other pHGG vaccine modalities remain in the preclinical stage with encouraging preliminary data. However, numerous challenges remain, including the identification of optimal antigen targets, overcoming the barriers imposed by CNS relative immune privilege and glioma-mediated immunosuppression, and ensuring timely vaccine delivery given the rapid progression of pHGG. Additionally, ethical and logistical considerations in the pediatric population require careful navigation, particularly when pursuing highly individualized experimental therapies in patients with limited therapeutic alternatives.

This review summarizes the current landscape of vaccine-based immunotherapy for pHGG, including peptide, dendritic cell (DC), mRNA, shared mutant neoantigen, and personalized neoantigen vaccine therapies. We outline the scientific rationale for vaccine development in this population, review key tumor antigens and immunologic barriers, and evaluate the major vaccine platforms under clinical investigation.

## Rationale for vaccine immunotherapy in pediatric HGG

**Fig. 1:**
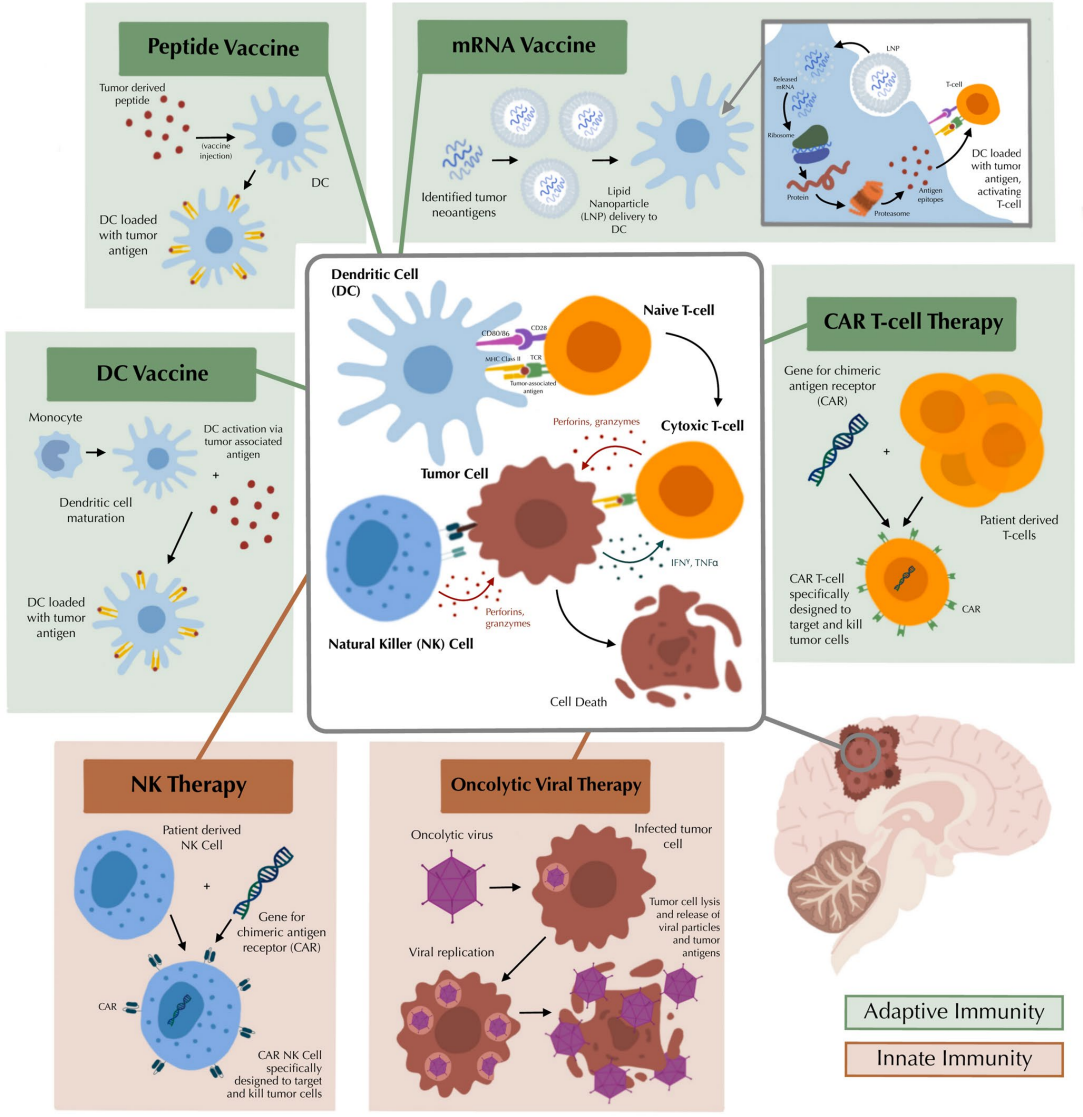
Immunotherapeutic strategies under investigation for pediatric high-grade gliomas. Adaptive immune therapies include peptide vaccines, mRNA vaccines, dendritic cell (DC) vaccines and chimeric antigen receptor (CAR) T-cell therapies. Innate immune therapies include natural killer (NK) cell therapy and oncolytic viral therapy

Given the dismal prognosis and resistance to conventional therapies, novel treatment strategies for pHGG are critical. Advances in modern sequencing techniques and growing insight into how histone-based mutations drive tumorigenesis have enabled the development of targeted molecular approaches [27, 28]. Among these, vaccine-based therapies harness the body’s immune system to recognize and attack TAAs. Although still investigational, early-phase clinical trials of multiple vaccine platforms have demonstrated immunogenicity and favorable safety profiles. A schematic representation of the various vaccine modalities currently under investigation is presented in Fig. 1. Current efforts are focused on refining antigen selection, enhancing delivery platforms, and evaluating synergistic combinations with other immunotherapeutic agents. Continued clinical investigation is essential to confirm the therapeutic potential of pHGG vaccines in larger patient cohorts and to translate promising early findings into meaningful clinical benefit.

## Tumor antigens and potential vaccine targets

### Considerations for antigen selection

Antigen selection for vaccine therapies is predicated upon the identification of TAAs or neoantigens that are differentially expressed by tumor cells compared to healthy brain parenchyma. Comprehensive high-throughput genomic and proteomic sequencing enables the discovery of antigens, which must then be evaluated for major histocompatibility complex (MHC) binding affinity and potential to elicit immunogenic T-cell responses [29].

Several critical considerations guide the selection of suitable antigens. First, candidate antigens must be abundantly and consistently overexpressed in tumor cells relative to normal tissue, ensuring sufficient antigen presence to elicit a robust immune response. Antigens that are minimally expressed in normal, healthy tissues are preferred to reduce the risk of off-target autoimmune effects and to enhance therapeutic specificity. Second, antigens should demonstrate strong immunogenicity, indicating their capacity to provoke an effective T-cell-mediated immune response. Third, selected TAAs should ideally exhibit high prevalence across patient populations to ensure broad applicability. Tumor-specific neoantigens derived from unique somatic mutations may offer heightened specificity and avoid off-target effects; however, their widespread applicability could be limited given tumor heterogeneity across patients and pHGG subtypes. Lastly, antigens implicated in poor prognosis or therapy resistance are particularly attractive as their immunologic targeting may not only induce tumor regression but also disrupt pathways involved in tumor progression. Given the limited number of eligible patients and the poor prognosis of pHGG, only the most promising and rigorously validated antigens must be advanced into clinical trials to maximize the potential for therapeutic success and minimize risk in this vulnerable population.

### TAAs in Glioma

TAAs identified in gliomas represent a group of frequently overexpressed or uniquely mutated proteins that are conserved across many pHGG patients. This makes them appealing targets for vaccine-based therapies given their wide applicability across the population. Prominent TAAs currently under investigation include EphA2, IL-13Rα2, and survivin [22, 23]. These antigens are prioritized based on consistent overexpression in glioma cells relative to normal tissue, demonstrated capacity to induce antigen-specific T-cell responses, and established safety profiles in preliminary clinical studies [30, 31]. Beyond these well-established antigens, emerging mRNA-based vaccine strategies have expanded the repertoire of targetable antigens, encompassing proteins such as TP53, IDH1, C3, TCF12, ANXA5, FKBP10, MSN, and PYGL [32, 33]. These candidate antigens have been identified through integrative genomic and immunologic analyses as correlating strongly with poor prognosis and immune cell infiltration, underscoring their potential clinical significance in vaccine formulations for pHGG.

### Neoantigens Derived from Somatic Mutations

Neoantigens, derived from tumor-specific somatic mutations, represent highly specific targets for vaccine therapies and can be broadly categorized into shared mutant antigens (arising from recurrent cancer-specific mutations) or personalized neoantigens (patient-specific genetic alterations). Because neoantigens are not present in normal tissues, they bypass central immune tolerance and reduce the risk of autoimmunity. Among pHGG, the most extensively studied shared mutant neoantigen is the H3.3K27M mutation, a hallmark alteration in DMG [34, 35]. This mutation generates a unique and immunologically distinct peptide sequence which is absent in healthy tissues, making it an attractive target for antigen-specific vaccine development. Additional shared mutant neoantigens, including recurrent mutations in IDH1 and TP53, have also emerged as potential vaccine targets [32, 35]. In contrast, personalized neoantigens arise from unique, patient-specific somatic mutations and give rise to de novo peptide sequences that vary between patients. These personalized neoantigens form the basis for individualized neoantigen vaccine strategies. Together, neoantigen targets highlight the potential of personalized vaccine strategies to elicit robust, tumor-specific immune responses in pHGG while avoiding off-target autoimmune effects.

## Immunologic challenges in pediatric brain tumors

The CNS exhibits an immune landscape fundamentally distinct from the rest of the body, characterized by tightly regulated immune responses to protect neural tissue from inflammatory damage. While the brain has historically been described as immune privileged, this privilege is now understood to be relative rather than absolute, as activated T-cells are capable of trafficking across the BBB and mounting immune responses under physiological and therapeutic conditions such as systemic immunotherapy [36, 37]. Nevertheless, CNS immune responses remain tightly regulated through the BBB, specialized lymphatic drainage, and immunoregulatory mechanisms at CNS interfaces such as the meninges and perivascular spaces. While immune surveillance does occur, robust immune response within the parenchyma is limited to prevent collateral damage to neural tissue with limited regenerative capacity [36, 38]. As a result, immune-based therapies in the CNS (particularly in critical midline structures such as the brainstem) carry a risk of immune-mediated pseudo-progression. In these regions with limited tolerance for treatment-induced inflammation and edema, this phenomenon can mimic tumor progression, lead to worsening neurologic compromise and necessitate urgent administration of corticosteroids [22]. Therefore, therapeutic strategies aiming to stimulate antitumor immunity in the CNS must overcome these barriers to enable effective immune cell infiltration and activation within the tumor microenvironment.

In addition, as part of their resistance mechanism, pHGGs actively create an immunosuppressive microenvironment that inhibits effective antitumor immunity. This environment is marked by low cytotoxic T-cell infiltration, abundant immunosuppressive myeloid populations, and secretion of inhibitory cytokines like TGF-β and IL-10 [13, 36]. Single-cell transcriptomics have demonstrated that myeloid cells represent the predominant immune population in pHGG, yet their composition and functional states are distinct from those observed in adult glioblastoma and vary according to tumor location and histone mutations [6, 39, 40]. Pediatric gliomas, particularly H3.3K27M DMG, are enriched for heterogeneous disease-associated myeloid populations that exhibit impaired interferon signaling, limited antigen presenting capacity, and express chemokines to recruit additional suppressive myeloid cells and restrict lymphocyte infiltration [39]. Within this myeloid compartment, resident microglia and infiltrating bone-marrow derived macrophages (BMDMs) play complementary roles. Microglia preferentially localize to tumor margins and perivascular regions, whereas BMDMs infiltrate the tumor core and actively suppress T-cell proliferation [39, 41, 42]. Together, this myeloid landscape restricts T-cell access to the tumor core and suppresses their function, providing a possible mechanistic explanation for limited intratumoral immune activity despite detectable peripheral vaccine responses.

Another challenge is the low tumor mutational burden seen in pHGGs. Compared to many tumor types seen in adults, which are generally thought to develop over years as numerous somatic mutations accumulate, pHGGs exhibit a characteristically low mutational burden and instead arise from developmental and epigenetic alterations. This scarcity of somatic mutations limits the number of neoantigen targets for vaccine therapies as fewer TAAs are available for immune recognition [6, 14, 43].

Aside from the tumor-specific challenges, there are also challenges inherent to the pediatric population. For example, the pediatric immune system is characterized by a higher proportion of naïve T-cells and fewer memory T-cells and thus presents unique challenges for vaccine therapies. These less-experienced immune systems have reduced capacity for robust and durable adaptive responses to novel antigens [44]. When compounded by the relative immune privilege of the CNS and an immunosuppressive tumor microenvironment, the efficacy of vaccine-based interventions may be severely limited. In addition, emerging evidence suggests that T-cell dysfunction in pHGG differs from the classical T-cell exhaustion observed in adult glioblastoma. Whereas glioblastoma is characterized by profound T-cell exhaustion with upregulation of immune checkpoint molecules and clonal T-cell expansion [45], pHGG instead exhibit sparsely infiltrating T-cells in immature states, limited clonal expansion, and reduced effector functionality [40, 42]. These differences suggest that immune failure in pHGG may reflect impaired T-cell priming and recruitment rather than exhaustion alone.

This challenge is further compounded by steroid therapy, which is frequently used to manage cerebral edema in pHGG patients. Unfortunately, although beneficial for edema, steroids suppress immune function, resulting in reduced lymphocyte counts and impaired antigen presentation. Coupled with lymphopenia resulting from tumor progression and conventional therapies including temozolomide and radiation therapy, these immunosuppressive effects greatly impair vaccine-induced T-cell responses [21, 24]. The decision to offer steroids must be carefully considered in patients eligible for vaccine-based therapies, weighing the dangers of worsening cerebral edema against a possible reduction in vaccine efficacy. Future clinical investigations should rigorously monitor if patients are concurrently receiving steroid treatment to better evaluate if steroid use is contraindicated in pHGG vaccines.

## Vaccine strategies, trial outcomes, and biomarkers of response

### Peptide-based vaccines

Peptide-based vaccines utilize short amino acid sequences corresponding to epitopes derived from TAA to elicit an immune response against cancer cells expressing the chosen antigen. Upon administration, these peptides are internalized by antigen-presenting cells (APCs) and presented on the cell surface by MHC molecules. This activates antigen-specific CD8⁺ cytotoxic T lymphocytes or CD4⁺ helper T-cells, thereby initiating a targeted immune response [21–24]. Peptide-based vaccines currently under investigation for pHGG target both shared TAA and tumor-specific neoantigens.

#### H3.3K27M peptide vaccine

The H3.3K27M_26 − 35_ peptide vaccine is an investigational therapy targeting the H3.3K27M mutation characteristic of DMG. A phase I trial (NCT02960230) evaluating this vaccine enrolled 29 children with H3.3K27M-positive DMG and demonstrated that the vaccine was well tolerated without severe immune-related adverse events (Table 1) [21]. Patients who mounted an H3.3K27M-specific CD8⁺ T-cell response exhibited significantly improved median overall survival (OS) compared to non-responders. However, concurrent corticosteroid use was associated with diminished CD8⁺ T-cell responses, potentially blunting therapeutic efficacy. While this vaccine was initially investigated in patients with the human leukocyte antigen (HLA) subtype A02:01, emerging analyses suggest that immune responses to this vaccine can involve multiple HLA class II alleles and B-cell activation, indicating broader immunogenic potential.

**Table 1:** Summary of clinical trials evaluating active immunotherapies in pediatric high-grade glioma

Vaccine Type	Trial #	Paper Title	Population	Target Antigen	Delivery Method	Adjuvants	Number of Patients	Immune Response	PFS	OS	Safety Profile
Peptide Vaccine (H3.3K27M)	NCT02960230	Mass cytometry detects H3.3K27M-specific vaccine responses in diffuse midline glioma [21]	Age 3–21 years, newly diagnosed DIPG or DMG. HLA-A*02:01 + and H3.3K27M+	H3.3K27M_26–35_	Subcutaneous every 3 weeks for first 8 doses, then every 6 weeks. Maximum treatment of 96 weeks total	Tetanus Toxoid (TT) peptide, poly-ICLC and Montanide-ISA 51 VG	29 (19 DIPG, 10 DMG)	39% had H3.3K27M-reactive CD8⁺ T cell expansion	4.9 mo (DIPG), 3.5 mo (DMG). Immune responders had greater PFS than non-responders (*p* = 0.05)	Median 16.1 months (immune responders) vs. 9.8 months (non-responders), *p* = 0.05; overall 12-month OS ~ 40%	Grade 1–2 AEs, injection site reactions most common. One case of suspected aseptic meningitis (resolved)
Peptide Vaccine (H3K27M long peptide)	Compassionate use basis; ongoing INTERCEPT-H3 trial: NCT04808245	A H3K27M-targeted vaccine in adults with diffuse midline glioma [46]	Adults > 18 years with progression of histologically-confirmed H3K27M^+^ DMG after standard radiotherapy and chemotherapy with TMZ	H3K27M_14–40_	Subcutaneous injection bi-weekly for 6 weeks, then monthly for 4 months, followed by quarterly thereafter	Montanide ISA-50; topical imiquimod	8 (all H3K27M^+^ DMG)	H3K27M-specific CD4^+^ T cell-dominated immune response in 5/8 patients (62.5%) by IFN-γ ELIspot. One patient developed pseudoprogression followed by sustained complete remission.	Median: 6.2 months from first vaccination	Median: 12.8 months from first vaccination One patient with sustained complete remission for > 31 months	No regimen-limiting toxicity. Grade 1 injection site reactions in 25%. One patient developed pseudoprogression. No grade 3 or higher vaccine-related AEs
Peptide Vaccine (GAA)	NCT01130077	Antigen-specific immune responses and clinical outcome after vaccination with glioma-associated antigen peptides and polyinosinic-polycytidylic acid stabilized by lysine and carboxymethylcellulose in children with newly diagnosed malignant brainstem and nonbrainstem gliomas [22]	Age 1–21 with high grade glioma and HLA-A2+	EphA2, IL-13Rα2, and survivin	Subcutaneous injection every 3 weeks for 8 doses, then doses every 6 weeks for up to 2 years	Tetanus Toxoid (TT) peptide, poly-ICLC and Montanide ISA-51	26 (20 brainstem glioma, 6 other high grade glioma)	62% of patients had immune response to at least one GAA epitope	Unspecified, 5 patients with pseudoprogression (19%)	Median 13.3 months for total cohort, 25.1 months among HGG patients	Grade 1 and 2 AEs, including injection-site reactions (100%), flu-like symptoms (92%), grade 1 GI symptoms (31%), grade 1 leukopenia (15%)
Peptide Vaccine (GAA)	NCT01130077	Antigen-specific immunoreactivity and clinical outcome following vaccination with glioma-associated antigen peptides in children with recurrent high-grade gliomas: Results of a pilot study [23]	Age 1 to < 22 with high grade glioma and HLA-A2+	EphA2, IL-13Rα2, and survivin	Subcutaneous injection every 3 weeks for 8 doses, then doses every 6 weeks for up to 2 years	Tetanus Toxoid (TT) peptide, poly-ICLC and Montanide ISA-51	12 (6 glioblastoma, 5 AA, 1 malignant gliomatosis cerebri)	9 of 10 evaluable patients (90%) had immune reactivity to at least one GAA	Median 4.1 months. 33% reached 6 months PFS.	Median OS: 12.9 months. 73% reached 6 months OS	Grade 1 and 2 AEs, including injection-site reactions (100%) and flu-like symptoms (100%), grade 1 GI symptoms (42%), grade 1 anemia (8%) and grade 1 leukopenia (8%)
Peptide vaccine (PEP-CMV)	NCT03299309	A peptide vaccine targeting the CMV antigen pp65 in children and young adults with recurrent high-grade glioma and medulloblastoma: a phase 1 trial [24]	Age > 18, recurrent medulloblastoma or malignant glioma	CMV pp65 (PEP-CMV). Synthetic long peptide (SLP) of 26 amino acids encoding epitopes of pp65	Intradermal injection of PEP-CMV every 2 weeks for first 3 doses, then every month. Maximum treatment of 10 years	PEP-CMV injection given in Montanide ISA-51 following Td toxoid preconditioning and 5-day temozolomide course to induce lymphopenia	36 (HGG patients who received ≥ 1 vaccine)	ELISpot in 21 patients with known CMV status: 76.2% (16/21) showed increased IFN-γ pp65-directed T cell activity	2.5 months (95% CI: 2.2–3.2)	6.5 months (95% CI: 4.6–8.4)	17 grade 1 AEs, 15 grade 2, 1 grade 3 (pyramidal tract syndrome), 1 grade 4 (cerebral edema). Most common AE was grade 1 injection site reaction (81%)
Dendritic cell (DC) Vaccine	None	Adjuvant dendritic cell-based tumour vaccination for children with malignant brain tumours [25]	Children with malignant brain tumor	Autologous tumor lysate	Intradermal injections of DCs pulsed with tumor lysate via 4 distinct schedules	Topical imiquimod one night prior and 2 nights following each vaccination	45 (33 HGG, 5 MB/PNET, 4 ependymoma, 3 ATRT)	Of 23 tested patients, 19 (83%) showed vaccine-specific T-cell proliferation; 6 patients had > 24 months survival	Median 4.4 months. 42% reached 6 months PFS	Median OS: 13.5 months; 6 patients > 24 months survival	Only mild vaccine-related adverse events: fever, local skin reactions, fatigue, vomiting, headache
Dendritic cell (DC) Vaccine	NCT00107185	Autologous tumor lysate-pulsed dendritic cell immunotherapy for pediatric patients with newly diagnosed or recurrent high-grade gliomas [26]	Age 1–18 with confirmed WHO grade III or IV glioma	Autologous tumor lysate	Intradermal injection biweekly. Patients received between 2–4 doses	None	3 (2 glioblastoma, 1 anaplastic oligoastrocytoma)	Mild response of Th1/Th2 cytokines including IFN-γ, IL-2, IL-4, IL-6, TNFα	Unspecified.	Two patients survived at least 51 and 40 months following surgery. Last patient had 9 month OS	Mild adverse events (headache, injection-site erythema). One Grade 4 reaction with elevation of alkaline phosphatase (resolved)
Personalized Neoantigen Peptide Vaccine	None	A real-world observation of patients with glioblastoma treated with a personalized peptide vaccine [47]	Age 9–87 years; histologically-confirmed IDH-wildtype glioblastoma	Patient-specific tumor neoantigens (median 19 peptides per patient)	Intracutaneous injection 4 times in first 2 weeks, with subsequent booster vaccinations every 4–6 weeks	Sargramostim (GM-CSF) injection; topical imiquimod on injection site	173 (70 primary IDH-wt GBM, 103 recurrent IDH-wt GBM)	Neoantigen specific T cell responses were detected in 87/97 evaluable patients (90%).	Immunological responders Median: 28.7 months Non-responders median: 17.4 months. HR 0.47 (0.24–0.94), *p* = 0.03	Median 31.9 months from diagnosis	AE predominantly grade 1–2. Four grade 3 reactions (allergic reaction, anaphylaxis, skin reaction). No grade 4 AE observed.
CAR T cell (B7-H3)	NCT04185038	Intracerebroventricular B7-H3-targeting CAR T cells for diffuse intrinsic pontine glioma: a phase I trial [17]	Age 1–26 years with high grade glioma or diffuse midline glioma H3K27M-altered following standard radiation therapy	B7-H3 (CD276)	Repetitive intracerebroventricular (ICV) infusion every 2 weeks for 8 weeks with escalating doses, then continued dosing every 2–4 weeks	None; no lymphodepletion	21 (17 DMG via molecular diagnosis, 1 pontine anaplastic astrocytoma, 3 suspected DMG via radiographic diagnosis)	CAR T detected in CSF in 13/18 tested patients (72%). CSF elevation of CXCL10, IP-10 and GM-CSF	Unspecified. 9 patients were enrolled before progression, 12 patients enrolled after progression.	Median survival from first CAR T cell infusion: 10.7 months Median survival from diagnosis: 19.8 months 3 long term survivors > 44 months	Mostly grade 1–2 adverse events (most common: headache, nausea/vomiting, fatigue, fever). One grade 4 intratumoral hemorrhage
CAR T cell (GD2)	NCT04196413	Intravenous and intracranial GD2-CAR T cells for H3K27M^+^ diffuse midline gliomas [18]	Age 2–30 years with biopsy-confirmed H3K27M-mutant DMG, including DIPG or spinal DMG, who had completed standard radiotherapy	GD2	Single IV infusion of GD2-CAR T cells, followed by sequential IV or ICV infusions for patients with radiographic or clinical benefit	Lymphodepleting chemotherapy prior to IV infusion; no lymphodepletion for ICV infusions	13 enrolled; 11 treated (9 DIPG, 2 spinal DMG)	Peripheral expansion and persistence of GD-2 CR T cells in blood and CSF with repeat ICV infusions. 8/11 patients classified as “responders”.	Tumor volume reduction was observed in 7/11 patients, with > 50% reduction in 4 patients.	Median survival from diagnosis: 20.6 months 2 long term DIPG survivors (30, 33 months)	All patients (11/11) experienced CRS following initial IV GD2-CAR T infusion, DLT at higher dose level (3 patients with grade 4 CRS). No DLT with ICV infusion observed. TIAN observed in 71% of ICV infusions.

In parallel, important insights have emerged from adult studies utilizing the long 27-mer H3K27M_14 − 40_ peptide vaccine in the INTERCEPT H3 trial (NCT04808245). In a compassionate-use cohort of eight adult DMG patients, vaccination was well-tolerated with only grade 1 adverse events, and the vaccine induced mutation-specific immune responses detectable in both peripheral blood and cerebrospinal fluid [48]. Subsequent in-depth immune profiling of a single patient demonstrated that the 27-mer H3K27M peptide resulted in a robust CD4^+^ T-cell response with polyclonal T-cell receptor expansion across multiple HLA alleles [46]. While these findings are derived from adult DMG patients and cannot be directly extrapolated to pediatric populations, they provide evidence that H3K27M peptide vaccines can induce coordinated adaptive immune responses.

However, recent work by Wang et al. challenges the feasibility of this approach [49]. Using patient-derived H3.3K27M DMG models, they demonstrated that the H3.3K27M_26–35_ peptide is not endogenously presented at detectable levels on HLA-A02:01⁺ tumor cells. Mass spectrometry failed to identify the peptide-HLA complex on multiple diffuse intrinsic pontine glioma (DIPG) cell lines and engineered T-cells specific for H3.3K27M-HLA-A*02:01 failed to kill patient-derived tumor cells. These findings suggest that, despite in vitro binding capacity, the absence of natural peptide presentation may render vaccines or T-cell therapies targeting this epitope ineffective. Further investigation is ongoing.

#### TAA peptide vaccine

TAA peptide vaccines targeting EphA2, IL-13Rα2, and survivin have demonstrated safety and modest immunogenicity in pHGG (NCT01130077, Table 1) [22]. There were no observed dose-limiting toxicities, and results of enzyme-linked immunosorbent spot analysis (ELISA) demonstrated the TAA peptide vaccine was able to induce antigen-specific T-cell responses in 62% of patients to at least one of the three TAA epitopes. Immunologic pseudo-progression was reported in five patients (19%), who required corticosteroid management and cessation of subsequent TAA vaccine, although some patients were able to resume at a lower poly-ICLC dose after symptomatic resolution [22]. Another cohort study of twelve children with recurrent pHGG from the same trial (NCT01130077) showed median progression-free survival (PFS) and OS were 4.1 and 12.9 months, respectively. This study demonstrated only grade 1 and 2 adverse events with a favorable safety profile, and 90% of evaluable patients had TAA immune reactivity (Table 1) [23].

#### CMV pp65 (PEP-CMV) peptide vaccine

Human cytomegalovirus (CMV) epitopes have been found in 67% of pediatric high-grade gliomas, with prior work utilizing a dendritic cell vaccine showing modest efficacy [24]. The PEP-CMV pp65 peptide vaccine is a 26-amino-acid chain that targets the CMV pp65 antigen on pHGG cells. Notably, this antigen is absent in healthy brain tissue. A phase I trial (NCT03299309) showed the safety of the CMV pp65 vaccine with mostly grade 1–2 adverse events, although rare severe CNS toxicities such as cerebral edema were reported. The vaccine elicits pp65-specific T-cell responses in 76% of patients with a median PFS of 2.5 months and OS of 6.5 months (Table 1) [24]. A multi-institutional phase II trial (NCT05096481) is ongoing to evaluate efficacy and immunological response in a larger cohort.

Notably, the presence of CMV within gliomas remains an area of ongoing controversy. While multiple groups have reported detection of CMV proteins or nucleic acids in glioma specimens using immunohistochemistry or PCR-based approaches [50, 51], others have failed to detect CMV [52, 53]. These discrepancies have raised concerns regarding assay sensitivity, specificity and reproducibility. Importantly, even in the absence of productive viral replication, low-level CMV antigen expression has been proposed as a potential immunotherapeutic target [53], providing a rationale for continued investigation of CMV peptide vaccines despite this ongoing controversy.

In summary, peptide vaccines targeting H3.3K27M, shared TAA (EphA2, IL-13Rα2, survivin), and CMV pp65 are actively under clinical investigation for pHGG. Early-phase trials consistently demonstrate safety and modest immune response generation in over half of patients, though efficacy data remain to be validated in larger, controlled studies.

### Dendritic cell vaccines

Dendritic cell (DC) vaccines leverage the antigen-presenting function of dendritic cells to stimulate immune responses against high-grade gliomas. Patient-derived monocytes are differentiated, loaded with tumor antigens, and administered subcutaneously or intradermally. These DCs migrate to draining lymph nodes, activating CD8⁺ cytotoxic and CD4⁺ helper T-cells [25, 26]. This approach aims to overcome glioma-induced immune suppression by generating robust, tumor-specific immunity.

While DC vaccines have undergone extensive evaluation in the adult glioblastoma population, few trials evaluate their efficacy in the pHGG population. Ardon et al. demonstrated the feasibility of autologous tumor-lysate loaded DC vaccines in 45 children with relapsed malignant brain tumors, including 33 with pHGG [25]. Their study reported a median OS of 13.5 months for the HGG cohort, with six long-term survivors exceeding 24 months. DC vaccination was well tolerated, with only mild adverse events (Table 1). The ADDICT-pedGLIO (NCT04911621) trial is an ongoing investigation into a promising evolution of DC vaccinations utilizing Wilms’ tumor 1 (WT1) mRNA, an antigen overexpressed in pHGG including DIPG. This Phase I trial will establish safety and feasibility of DC vaccine leukapheresis protocols targeting the WT1 antigen in 10 children.

Lasky et al. conducted tumor lysate-pulsed DC vaccination in three children with high grade glioma (NCT00107185) [26]. They showed a favorable safety profile and only mild adverse events, including one patient who had a transient, asymptomatic elevation of alkaline phosphatase. Two patients had extended PFS of at least 51 and 40 months (Table 1). This study also underscored the significant feasibility challenges in DC vaccine administration, as rapid disease progression precluded vaccine administration in four patients. Trials investigating alternative splice variant-targeted peptide-pulsed DCs (NCT06342908) and the CMV pp65-LAMP mRNA-pulsed DCs (NCT03688178) are ongoing.

Despite encouraging safety profiles and signals of potential efficacy in select patients, DC vaccines face significant challenges in pHGG, including lengthy manufacturing times that can delay treatment in rapidly progressing disease, variability in immune responsiveness, and the profound immunosuppressive tumor microenvironment that may limit durable antitumor activity. Additionally, limited patient numbers in early-phase trials limit definitive conclusions regarding survival benefit. Future studies are critical to refine antigen selection, optimize timing of vaccine delivery, and explore synergistic combinations with other immunomodulatory or standard therapies, including radiation.

### mRNA-based vaccines

Messenger RNA (mRNA) vaccines represent another therapeutic approach utilizing the patient’s adaptive immune system to target malignant cells. Unlike peptide vaccines, delivery of mRNA sequences encoding TAAs allows APCs to translate the full-length antigen and present multiple epitopes on MHC molecules without restriction to only certain HLA types [54]. This allows for a more robust immune response by stimulating cytotoxic CD8⁺ T lymphocytes and helper CD4⁺ T-cells through antigen presentation from both MHC-I and MHC-II molecules. Ex vivo loading of patient-derived DCs with mRNA encoding glioma antigens allows APCs to prime naïve T-cells upon reinfusion. Alternatively, direct injection of lipid nanoparticle (LNP)-encapsulated mRNA enables in situ translation and immune activation without ex vivo processing [55]. These strategies enable the immune system to mount a multi-epitope response with low rates of immune rejection and no risk of genome integration, offering unique advantages compared to other vaccine modalities.

Several TAA have been identified as possible targets for mRNA-based vaccines for gliomas including TP53, C3, TCF12, and IDH-1 [29, 32]. A comprehensive analysis of glioma datasets from The Cancer Genome Atlas (TCGA) and the Chinese Glioma Genome Atlas (CGGA) identified these antigens as overexpressed and correlated with increased infiltration of APCs, suggesting their potential utility as targets in mRNA-based vaccines. Studies have also noted the expression of immunosuppressive molecules such as PD-L1 and TIM-3 in pHGGs, supporting the rationale for combining vaccines with immune checkpoint blockade to enhance antitumor response [56, 57]. However, checkpoint inhibition alone has demonstrated only limited clinical success to date in pHGG, underscoring that any potential synergistic approach with vaccine therapies will require careful clinical validation.

Clinical translation of mRNA vaccines specifically for pediatric gliomas remains in its infancy, with no completed trials reported in children to date. However, adult trials targeting similar antigens, including early-phase studies using mRNA-loaded DC vaccines and direct LNP-encapsulated mRNA delivery, have demonstrated safety and immunogenicity, laying the groundwork for currently ongoing pediatric trials [58]. Ongoing identification of glioma antigens and evolving understanding of the unique pediatric tumor immune microenvironment are critical for the success of this vaccine modality. Trials investigating the CVGBM mRNA vaccine (NCT05938387) and RNA-lipid Particle (RNA-LP) vaccines (NCT04573140) are ongoing.

### Neoantigen-personalized vaccines

Neoantigen-personalized vaccines represent a unique, highly targeted approach for immunotherapy in pHGG, driven by the unique genomic landscape of individual tumors. Unlike shared TAAs central to other glioma vaccine therapies, neoantigens arise from tumor-specific mutations, presenting entirely novel peptide sequences not found in healthy tissues that are specific to each *individual* patient [34]. In pediatric medulloblastoma, for instance, Rivero-Hinojosa et al. show aberrant splice junctions as significant neoantigen sources through proteogenomic analysis [59].

While pediatric trials utilizing neoantigen-personalized vaccines have not been conducted, early-phase clinical trials in adult glioblastoma have shown promising results. The personalized neoantigen vaccine “NeoVax”, which combines patient-specific neoantigens identified from tumor sampling, has successfully generated immune responses characterized by an increase in interferon-gamma (IFN-γ) producing T-cells and intratumoral T-cell infiltration [60]. In a cohort of 173 adult patients with IDH-wildtype glioblastoma, personalized multi-peptide neoantigen vaccines were feasible to manufacture (median time from tissue acquisition to vaccine administration was 16 weeks), well tolerated with only grade 1 or 2 adverse events, and induced durable T-cell responses in 77 of 97 patients tested [47]. Importantly, patients who were found to mount an effective immune response against multiple vaccinated neoantigens were associated with prolonged survival compared with low- or non-responders. These data provide important early evidence for the potential of neoantigen vaccines, albeit exclusively in an adult population. Although data specific to pHGG are limited at this time, the biological rationale and encouraging early-phase adult studies highlight the translational promise of neoantigen vaccines for pediatric populations, especially given the significant heterogeneity in pHGG mutations.

Despite the considerable potential, several challenges remain for neoantigen vaccine implementation. pHGGs typically possess fewer somatic mutations compared to adult tumors, which may complicate neoantigen identification and limit target availability [6, 13–15]. Furthermore, the tumor microenvironment in pediatric gliomas often demonstrates pronounced immunosuppression, reducing T-cell infiltration and activity, potentially undermining vaccine efficacy [13, 36]. Additionally, access to tissue samples for sequencing and neoantigen identification can be especially challenging in certain pHGG, depending on anatomical location and surgical accessibility. The requirement for patient-specific manufacturing also introduces logistical complexity, substantial costs, and potential delays that may be incompatible with the rapid progression of HGG. Given the rapid expansion of these tumor populations, the potential for intratumoral heterogeneity may also further complicate vaccine design [56]. Despite these physiologic and logistical challenges, the incorporation of combination approaches such as neoantigen vaccines alongside immune checkpoint inhibitors may enhance antitumor efficacy.

## Ethical, regulatory, manufacturing, and translational challenges

Developing vaccines for pHGG presents a host of challenges. In this vulnerable population, characterized by an aggressive disease course and limited treatment options, maintaining a favorable risk-benefit balance is essential. Protecting patient welfare requires thoughtful, transparent communication between providers and families. Additional services to support the patient and family should be involved early, including social work, child life specialists, psychiatry, spiritual or cultural support, and palliative care. In the adolescent population, assessing decision-making capacity and navigating informed consent add further complexity, requiring a sensitive and individualized approach.

Regulatory oversight plays a critical role, mandating rigorous safety evaluations and continuous monitoring of vaccine trials by institutional review boards and ethics committees. Manufacturing challenges are substantial, particularly for personalized neoantigen vaccines, which require tumor sequencing, epitope selection, strict quality control, sterile production protocols, and efficient logistical coordination. In practice, this process ranges from 10 to 16 weeks from tissue acquisition to vaccine availability, a timeline that may be incompatible with the rapid clinical progression in many pHGG patients. Given this narrow therapeutic window, delays in manufacturing or trial enrollment may render patients ineligible for treatment due to worsening neurologic decline or tumor progression [26].

Logistical and financial barriers also further limit clinical translation. Vaccine platforms remain primarily accessible only within clinical trials, and reimbursement for investigational or personalized vaccines outside of a trial setting is often unclear and logistically challenging to navigate. These constraints limit equitable access and disproportionately restrict availability to patients treated at large academic centers with specialized infrastructure to host these clinical trials. Translational barriers also include the limited predictive value of preclinical models, a lack of validated biomarkers to monitor immune response and tumor progression, and the inherent difficulties in designing and executing clinical trials for a rare disease with a small and often rapidly deteriorating patient population. Overcoming these multifaceted challenges is essential to ensure the safe, timely, and effective advancement of vaccine-based therapies for children with high-grade gliomas.

## Conclusions & future directions

Despite encouraging early-phase data, significant knowledge gaps remain in understanding how vaccine therapies can be optimized for pHGG. Key uncertainties include the most effective antigen targets for broad or personalized application, the optimal timing and sequencing of vaccine administration relative to standard therapies, and the impact of immune-modulating factors such as steroid use on vaccine efficacy. Moreover, the pediatric-specific immune response to tumor vaccines remains poorly characterized, particularly in the setting of CNS relative immune privilege and glioma-mediated suppression. The lack of validated biomarkers to predict or monitor treatment response further complicates trial design and clinical implementation.

An additional priority for future investigation is defining the optimal clinical windows for vaccine administration as pHGG treatment continues to evolve. Vaccine-based therapies may be the most effective during periods of minimal disease burden, such as following maximal safe surgical resection or in the post-radiation setting, when tumor antigen release and blood-brain barrier disruption may enhance immune priming. In contrast, concurrent administration during upfront chemoradiation may be limited by treatment-induced lymphopenia and the frequent requirement for corticosteroids. The recent approval of ONC201 for H3.3K27M-mutant DMG may also introduce a new therapeutic axis and expand the window of opportunity to maximize response to vaccine therapies by enabling disease stabilization with a favorable toxicity profile and reduced steroid dependence, thereby creating more favorable conditions for vaccine integration.

Future research must prioritize the development of integrated pipelines to rapidly identify high-quality tumor antigens in individual patients given the rapid progression of pHGG. Studies exploring combination therapies, such as vaccines with radiation or immune checkpoint inhibitors, are essential to enhance immunogenicity and overcome tumor-induced suppression or intratumoral heterogeneity. Pediatric-focused clinical trials with biomarker-correlated endpoints and immune profiling will be critical to refine dosing, timing, and patient inclusion criteria. Advancements in delivery platforms, such as focused ultrasound-assisted delivery across the BBB, may further increase therapeutic penetration and efficacy.

Vaccine-based immunotherapy represents a promising yet still nascent strategy in the treatment of pediatric high-grade gliomas. While initial trials have demonstrated safety and immunogenic potential, clinical efficacy has yet to be conclusively proven. Addressing the biological, logistical, and ethical challenges unique to this population will require sustained multidisciplinary collaboration across institutions. With continued innovation and rigorous clinical validation, vaccine therapies may one day complement or even transform the therapeutic landscape for children facing these devastating tumors.

## Data Availability

No datasets were generated or analysed during the current study.

## References

[1] Cohen AR (2022) Brain tumors in children. N Engl J Med 386:1922–1931. 10.1056/NEJMra2116344 35584157 10.1056/NEJMra2116344

[2] Maxwell R, Luksik AS, Garzon-Muvdi T et al (2018) Population-based study determining predictors of Cancer-Specific mortality and survival in pediatric High-grade brainstem glioma. World Neurosurg 119:e1006–e1015. 10.1016/j.wneu.2018.08.044 30138731 10.1016/j.wneu.2018.08.044

[3] Kallappagoudar S, Yadav RK, Lowe BR, Partridge JF (2015) Histone H3 mutations—a special role for H3.3 in tumorigenesis? Chromosoma 124:177–189. 10.1007/s00412-015-0510-4 25773741 10.1007/s00412-015-0510-4 PMC4446520

[4] Hassan H, Pinches A, Picton SV, Phillips RS (2017) Survival rates and prognostic predictors of high grade brain stem gliomas in childhood: a systematic review and meta-analysis. J Neurooncol 135:13–20. 10.1007/s11060-017-2546-1 28681244 10.1007/s11060-017-2546-1 PMC5658459

[5] Hargrave D, Bartels U, Bouffet E (2006) Diffuse brainstem glioma in children: critical review of clinical trials. Lancet Oncol 7:241–248. 10.1016/S1470-2045(06)70615-5 16510333 10.1016/S1470-2045(06)70615-5

[6] Ross JL, Velazquez Vega J, Plant A et al (2021) Tumour immune landscape of paediatric high-grade gliomas. Brain 144:2594–2609. 10.1093/brain/awab155 33856022 10.1093/brain/awab155 PMC8536940

[7] Gajjar A, Mahajan A, Abdelbaki M et al (2022) Pediatric central nervous system Cancers, version 2.2023, NCCN clinical practice guidelines in oncology. J Natl Compr Canc Netw 20:1339–1362. 10.6004/jnccn.2022.0062 36509072 10.6004/jnccn.2022.0062

[8] Miguel Llordes G, Medina Pérez VM, Curto Simón B et al (2023) Epidemiology, diagnostic Strategies, and therapeutic advances in diffuse midline glioma. J Clin Med 12:5261. 10.3390/jcm12165261 37629304 10.3390/jcm12165261 PMC10456112

[9] Armand P (2015) Immune checkpoint Blockade in hematologic malignancies. Blood 125:3393–3400. 10.1182/blood-2015-02-567453 25833961 10.1182/blood-2015-02-567453

[10] Wang H, Kaur G, Sankin AI et al (2019) Immune checkpoint Blockade and CAR-T cell therapy in hematologic malignancies. J Hematol Oncol 12:59. 10.1186/s13045-019-0746-1 31186046 10.1186/s13045-019-0746-1 PMC6558778

[11] Brudno JN, Maus MV, Hinrichs CS (2024) CAR T cells and T-cell therapies for cancer: a translational science review. 10.1001/jama.2024.19462. JAMA 332:1924 10.1001/jama.2024.19462 PMC11808657 39495525

[12] Kumar A, Emdad L, Das SK, Fisher PB (2024) Recent advances and progress in immunotherapy of solid cancers. Adv Cancer Res 164:111–190. 10.1016/bs.acr.2024.05.004 39306365 10.1016/bs.acr.2024.05.004

[13] Vetsika E-K, Katsianou MA, Sarantis P et al (2025) Pediatric gliomas immunity challenges and immunotherapy advances. Cancer Lett 618:217640. 10.1016/j.canlet.2025.217640 40090572 10.1016/j.canlet.2025.217640

[14] Persson ML, Douglas AM, Alvaro F et al (2022) The intrinsic and microenvironmental features of diffuse midline glioma: implications for the development of effective immunotherapeutic treatment strategies. Neuro Oncol 24:1408–1422. 10.1093/neuonc/noac117 35481923 10.1093/neuonc/noac117 PMC9435509

[15] Antonucci L, Canciani G, Mastronuzzi A et al (2022) CAR-T therapy for pediatric High-Grade gliomas: Peculiarities, current investigations and future strategies. Front Immunol 13:867154. 10.3389/fimmu.2022.867154 35603195 10.3389/fimmu.2022.867154 PMC9115105

[16] Hwang EI, Sayour EJ, Flores CT et al (2022) The current landscape of immunotherapy for pediatric brain tumors. Nat Cancer 3:11–24. 10.1038/s43018-021-00319-0 35121998 10.1038/s43018-021-00319-0

[17] Vitanza NA, Ronsley R, Choe M et al (2025) Intracerebroventricular B7-H3-targeting CAR T cells for diffuse intrinsic Pontine glioma: a phase 1 trial. Nat Med 31:861–868. 10.1038/s41591-024-03451-3 39775044 10.1038/s41591-024-03451-3 PMC11922736

[18] Monje M, Mahdi J, Majzner R et al (2025) Intravenous and intracranial GD2-CAR T cells for H3K27M + diffuse midline gliomas. Nature 637:708–715. 10.1038/s41586-024-08171-9 39537919 10.1038/s41586-024-08171-9 PMC11735388

[19] Faulhaber LD, Phuong AQ, Hartsuyker KJ et al (2022) Brain capillary obstruction during neurotoxicity in a mouse model of anti-CD19 chimeric antigen receptor T-cell therapy. Brain Commun 4:fcab309. 10.1093/braincomms/fcab309 35169706 10.1093/braincomms/fcab309 PMC8833245

[20] Belin C, Devic P, Ayrignac X et al (2020) Description of neurotoxicity in a series of patients treated with CAR T-cell therapy. Sci Rep 10. 10.1038/s41598-020-76055-9 10.1038/s41598-020-76055-9 PMC7642402 33149178

[21] Mueller S, Taitt JM, Villanueva-Meyer JE et al (2020) Mass cytometry detects H3.3K27M-specific vaccine responses in diffuse midline glioma. J Clin Invest 130:6325–6337. 10.1172/JCI140378 32817593 10.1172/JCI140378 PMC7685729

[22] Pollack IF, Jakacki RI, Butterfield LH et al (2014) Antigen-specific immune responses and clinical outcome after vaccination with glioma-associated antigen peptides and polyinosinic-polycytidylic acid stabilized by lysine and carboxymethylcellulose in children with newly diagnosed malignant brainstem and nonbrainstem gliomas. J Clin Oncol 32:2050–2058. 10.1200/JCO.2013.54.0526 24888813 10.1200/JCO.2013.54.0526 PMC4067943

[23] Pollack IF, Jakacki RI, Butterfield LH et al (2016) Antigen-specific immunoreactivity and clinical outcome following vaccination with glioma-associated antigen peptides in children with recurrent high-grade gliomas: results of a pilot study. J Neurooncol 130:517–527. 10.1007/s11060-016-2245-3 27624914 10.1007/s11060-016-2245-3 PMC5363717

[24] Thompson EM, Ashley DM, Ayasoufi K et al (2025) A peptide vaccine targeting the CMV antigen pp65 in children and young adults with recurrent high-grade glioma and medulloblastoma: a phase 1 trial. Nat Cancer. 10.1038/s43018-025-00998-z 40506525 10.1038/s43018-025-00998-z

[25] Ardon H, De Vleeschouwer S, Van Calenbergh F et al (2010) Adjuvant dendritic cell-based tumour vaccination for children with malignant brain tumours. Pediatr Blood Cancer 54:519–525. 10.1002/pbc.22319 19852061 10.1002/pbc.22319

[26] Lasky JL, Panosyan EH, Plant A et al (2013) Autologous tumor lysate-pulsed dendritic cell immunotherapy for pediatric patients with newly diagnosed or recurrent high-grade gliomas. Anticancer Res 33:2047–2056 23645755 PMC4018463

[27] Harutyunyan AS, Krug B, Chen H et al (2019) H3K27M induces defective chromatin spread of PRC2-mediated repressive H3K27me2/me3 and is essential for glioma tumorigenesis. Nat Commun 10:1262. 10.1038/s41467-019-09140-x 30890717 10.1038/s41467-019-09140-x PMC6425035

[28] Rakotomalala A, Bailleul Q, Savary C et al (2021) H3.3K27M mutation controls cell growth and resistance to therapies in pediatric glioma cell lines. Cancers (Basel) 13:5551. 10.3390/cancers13215551 34771714 10.3390/cancers13215551 PMC8583077

[29] Zhang JG, Eguchi J, Kruse CA et al (2007) Antigenic profiling of glioma cells to generate allogeneic vaccines or dendritic cell-based therapeutics. Clin Cancer Res 13:566–575. 10.1158/1078-0432.CCR-06-1576 17255279 10.1158/1078-0432.CCR-06-1576 PMC4030524

[30] Berlow NE, Svalina MN, Quist MJ et al (2018) IL-13 receptors as possible therapeutic targets in diffuse intrinsic Pontine glioma. PLoS ONE 13:e0193565. 10.1371/journal.pone.0193565 29621254 10.1371/journal.pone.0193565 PMC5886401

[31] Okada H, Low KL, Kohanbash G et al (2008) Expression of glioma-associated antigens in pediatric brain stem and non-brain stem gliomas. J Neurooncol 88:245–250. 10.1007/s11060-008-9566-9 18324354 10.1007/s11060-008-9566-9 PMC2561297

[32] Ma S, Ba Y, Ji H et al (2021) Recognition of Tumor-Associated antigens and immune subtypes in glioma for mRNA vaccine development. Front Immunol 12:738435. 10.3389/fimmu.2021.738435 34603319 10.3389/fimmu.2021.738435 PMC8484904

[33] Zhong H, Liu S, Cao F et al (2021) Dissecting tumor antigens and immune subtypes of glioma to develop mRNA vaccine. Front Immunol 12:709986. 10.3389/fimmu.2021.709986 34512630 10.3389/fimmu.2021.709986 PMC8429949

[34] Chheda ZS, Kohanbash G, Okada K et al (2018) Novel and shared neoantigen derived from histone 3 variant H3.3K27M mutation for glioma T cell therapy. J Exp Med 215:141–157. 10.1084/jem.20171046 29203539 10.1084/jem.20171046 PMC5748856

[35] Nejo T, Yamamichi A, Almeida ND et al (2020) Tumor antigens in glioma. Semin Immunol 47:101385. 10.1016/j.smim.2020.101385 32037183 10.1016/j.smim.2020.101385

[36] Smyth LCD, Kipnis J (2025) Redefining CNS immune privilege. Nat Rev Immunol 25:766–775. 10.1038/s41577-025-01175-0 40316862 10.1038/s41577-025-01175-0

[37] Yoshida TM, Wang A, Hafler DA (2022) Basic principles of neuroimmunology. Semin Immunopathol 44:685–695. 10.1007/s00281-022-00951-7 35732977 10.1007/s00281-022-00951-7

[38] Proulx ST, Engelhardt B (2022) Central nervous system zoning: how brain barriers Establish subdivisions for CNS immune privilege and immune surveillance. J Intern Med 292:47–67. 10.1111/joim.13469 35184353 10.1111/joim.13469 PMC9314672

[39] Ross JL, Puigdelloses-Vallcorba M, Piñero G et al (2024) Microglia and monocyte-derived macrophages drive progression of pediatric high-grade gliomas and are transcriptionally shaped by histone mutations. Immunity 57:2669–2687e6. 10.1016/j.immuni.2024.09.007 39395421 10.1016/j.immuni.2024.09.007 PMC11578068

[40] Cao L, Xie W, Ma W et al (2023) The unique immune ecosystems in pediatric brain tumors: integrating single-cell and bulk RNA-sequencing. Front Immunol 14:1238684. 10.3389/fimmu.2023.1238684 38094301 10.3389/fimmu.2023.1238684 PMC10716463

[41] Banerjee K, Ratzabi A, Caspit IM et al (2023) Distinct Spatiotemporal features of microglia and monocyte-derived macrophages in glioma. Eur J Immunol 53:e2250161. 10.1002/eji.202250161 36649079 10.1002/eji.202250161

[42] Messiaen J, Jacobs SA, De Smet F (2023) The tumor micro-environment in pediatric glioma: friend or foe? Front Immunol 14:1227126. 10.3389/fimmu.2023.1227126 37901250 10.3389/fimmu.2023.1227126 PMC10611473

[43] Friedman GK, Johnston JM, Bag AK et al (2021) Oncolytic HSV-1 G207 immunovirotherapy for pediatric High-Grade gliomas. N Engl J Med 384:1613–1622. 10.1056/nejmoa2024947 33838625 10.1056/NEJMoa2024947 PMC8284840

[44] Rosichini M, Del Baldo G, De Luca CD et al (2024) Pediatric brain tumor patients display altered immune activation and reduced lymphopoiesis at the onset of disease. NPJ Precis Oncol 8:269. 10.1038/s41698-024-00755-y 39567679 10.1038/s41698-024-00755-y PMC11579487

[45] Woroniecka K, Chongsathidkiet P, Rhodin K et al (2018) T-Cell exhaustion signatures vary with tumor type and are severe in glioblastoma. Clin Cancer Res 24:4175–4186. 10.1158/1078-0432.CCR-17-1846 29437767 10.1158/1078-0432.CCR-17-1846 PMC6081269

[46] Boschert T, Kromer K, Lerner T et al (2024) H3K27M neoepitope vaccination in diffuse midline glioma induces B and T cell responses across diverse HLA loci of a recovered patient. Sci Adv 10:eadi9091. 10.1126/sciadv.adi9091 38306431 10.1126/sciadv.adi9091 PMC10836722

[47] Latzer P, Zelba H, Battke F et al (2024) A real-world observation of patients with glioblastoma treated with a personalized peptide vaccine. Nat Commun 15:6870. 10.1038/s41467-024-51315-8 39127809 10.1038/s41467-024-51315-8 PMC11316744

[48] Grassl N, Poschke I, Lindner K et al (2023) A H3K27M-targeted vaccine in adults with diffuse midline glioma. Nat Med 29:2586–2592. 10.1038/s41591-023-02555-6 37735561 10.1038/s41591-023-02555-6 PMC10579055

[49] Wang SS, Pandey K, Watson KA et al (2023) Endogenous H3.3K27M derived peptide restricted to HLA-A∗02:01 is insufficient for immune-targeting in diffuse midline glioma. Mol Ther Oncolytics 30:167–180. 10.1016/j.omto.2023.08.005 37674626 10.1016/j.omto.2023.08.005 PMC10477804

[50] Cobbs CS, Harkins L, Samanta M et al (2002) Human cytomegalovirus infection and expression in human malignant glioma. Cancer Res 62:3347–3350 12067971

[51] Mitchell DA, Xie W, Schmittling R et al (2008) Sensitive detection of human cytomegalovirus in tumors and peripheral blood of patients diagnosed with glioblastoma. Neuro Oncol 10:10–18. 10.1215/15228517-2007-035 17951512 10.1215/15228517-2007-035 PMC2600830

[52] Holdhoff M, Guner G, Rodriguez FJ et al (2017) Absence of cytomegalovirus in glioblastoma and other High-grade gliomas by Real-time PCR, Immunohistochemistry, and in situ hybridization. Clin Cancer Res 23:3150–3157. 10.1158/1078-0432.CCR-16-1490 28034905 10.1158/1078-0432.CCR-16-1490 PMC5474132

[53] Lawler SE (2015) Cytomegalovirus and glioblastoma; controversies and opportunities. J Neurooncol 123:465–471. 10.1007/s11060-015-1734-0 25682092 10.1007/s11060-015-1734-0

[54] Mao M, Yang W, Zhang X (2024) Current mRNA-based vaccine strategies for glioma treatment. Crit Rev Oncol Hematol 202:104459. 10.1016/j.critrevonc.2024.104459 39097247 10.1016/j.critrevonc.2024.104459

[55] Karimi-Sani I, Molavi Z, Naderi S et al (2024) Personalized mRNA vaccines in glioblastoma therapy: from rational design to clinical trials. J Nanobiotechnol 22:601. 10.1186/s12951-024-02882-x 10.1186/s12951-024-02882-x PMC11453023 39367418

[56] Yoel A, Adjumain S, Liang Y et al (2024) Emerging and biological concepts in pediatric High-Grade gliomas. Cells 13:1492. 10.3390/cells13171492 39273062 10.3390/cells13171492 PMC11394548

[57] Ausejo-Mauleon I, Labiano S, de la Nava D et al (2023) TIM-3 Blockade in diffuse intrinsic Pontine glioma models promotes tumor regression and antitumor immune memory. Cancer Cell 41:1911–1926.e8. 10.1016/j.ccell.2023.09.001 37802053 10.1016/j.ccell.2023.09.001 PMC10644900

[58] Tabatabai G, Von Baumgarten L, Van Den Bent MJ et al (2024) Phase 1 dose-finding study to evaluate safety and tolerability of CVGBM in patients with newly diagnosed and surgically resected MGMT-unmethylated glioblastoma. JCO 42:TPS2095–TPS2095. 10.1200/jco.2024.42.16_suppl.tps2095

[59] Rivero-Hinojosa S, Grant M, Panigrahi A et al (2021) Proteogenomic discovery of neoantigens facilitates personalized multi-antigen targeted T cell immunotherapy for brain tumors. Nat Commun 12:6689. 10.1038/s41467-021-26936-y 34795224 10.1038/s41467-021-26936-y PMC8602676

[60] Keskin DB, Anandappa AJ, Sun J et al (2019) Neoantigen vaccine generates intratumoral T cell responses in phase Ib glioblastoma trial. Nature 565:234–239. 10.1038/s41586-018-0792-9 30568305 10.1038/s41586-018-0792-9 PMC6546179

